# Simultaneous Treatment with Statins and Aspirin Reduces the Risk of Prostate Cancer Detection and Tumorigenic Properties in Prostate Cancer Cell Lines

**DOI:** 10.1155/2015/762178

**Published:** 2015-01-12

**Authors:** M. Olivan, M. Rigau, E. Colás, M. Garcia, M. Montes, T. Sequeiros, L. Regis, A. Celma, J. Planas, J. Placer, J. Reventós, I. de Torres, A. Doll, J. Morote

**Affiliations:** ^1^Research Unit in Biomedicine and Translational Oncology, Vall d'Hebron Research Institute and Hospital and Autonomous University of Barcelona, Passeig Vall d'Hebron 119-129, 08035 Barcelona, Spain; ^2^Department of Urology, Vall d'Hebron University Hospital and Autonomous University of Barcelona, Passeig Vall d'Hebron 119-129, 08035 Barcelona, Spain; ^3^Departament de Ciències Bàsiques, Universitat Internacional de Catalunya, Josep Trueta s/n, Sant Cugat del Vallès, 08195 Barcelona, Spain; ^4^Bellvitge Biomedical Research Institute (IDIBELL), Gran Via de l'Hospitalet 199, 08907 Barcelona, Spain; ^5^Department of Pathology, Vall d'Hebron University Hospital and Autonomous University of Barcelona, Passeig Vall d'Hebron 119-129, 08035 Barcelona, Spain

## Abstract

Nowadays prostate cancer is the most common solid tumor in men from industrialized countries and the second leading cause of death. At the ages when PCa is usually diagnosed, mortality related to cardiovascular morbidity is high; therefore, men at risk for PCa frequently receive chronic lipid-lowering and antiplatelet treatment. The aim of this study was to analyze how chronic treatment with statins, aspirin, and their combination influenced the risk of PCa detection. The tumorigenic properties of these treatments were evaluated by proliferation, colony formation, invasion, and migration assays using different PCa cell lines, in order to assess how these treatments act at molecular level. The results showed that a combination of statins and aspirin enhances the effect of individual treatments and seems to reduce the risk of PCa detection (OR: 0.616 (95% CI: 0.467–0.812), *P* < 0.001). However, if treatments are maintained, aspirin (OR: 1.835 (95% CI: 1.068–3.155), *P* = 0.028) or the combination of both drugs (OR: 3.059 (95% CI: 1.894–4.939), *P* < 0.001) represents an increased risk of HGPCa. As observed at clinical level, these beneficial effects* in vitro* are enhanced when both treatments are administered simultaneously, suggesting that chronic, concomitant treatment with statins and aspirin has a protective effect on PCa incidence.

## 1. Introduction

Nowadays prostate cancer (PCa) is the most common solid tumor in men from industrialized countries and the second leading cause of death [1, 2]. For this reason, the discovery of modifiable risk determinants would provide an opportunity to prevent or delay the onset of this common disease. Established risk factors, such as ancestry or family history, are not modifiable, and the evidence for dietary and lifestyle risk factors is inconclusive [3].

At the ages when PCa is usually diagnosed, mortality related to cardiovascular morbidity is high [4]. Associations between coronary heart disease and PCa risk have even been suggested [5]; men at risk of PCa frequently receive chronic lipid-lowering and antiplatelet treatment [6, 7]. Statins and aspirin are two of the most frequently used drugs, and they both have been implicated in prostate carcinogenesis, albeit not without some controversy [8, 9].

Statins (3-hydroxy-3-methyl-glutaryl-coenzyme A reductase inhibitors) are the most commonly used drugs for lowering cholesterol [10]. In recent years, a growing amount of evidence on the anticancer effects of statins has become available from laboratory and epidemiological studies.* In vitro* and* in vivo* studies [11] suggest that statins are able to arrest cell cycle progression [12], induce apoptosis [13], reduce inflammation, and impede angiogenesis [14].

Currently, observational studies support that statin users have an unchanged overall risk of PCa; however, some well-designed epidemiological studies have reported a reduced incidence of advanced PCa in this population. Moreover, statins showed an inhibitory effect on the growth of PCa in both* in vitro* and* in vivo* studies [15]. On the other hand, in recent years the role of inflammation in cancer etiology has gained attention, and several studies have suggested that nonsteroidal anti-inflammatory drugs (NSAIDs) may have chemopreventive activity. NSAIDs have been associated with a reduced risk of detection for different malignancies: a reduction of the relative risk of 63% for colorectal cancer, 39% for breast cancer, 36% for lung cancer, and up to 39% for PCa [16]. In particular, the use of acetyl salicylic acid (ASA), the active metabolite of aspirin and a well-known NSAID, is associated with a 15%–55% risk reduction for PCa [17–20]; however, two other studies associated aspirin with increased risk of PCa [21, 22], while others reported no association [23, 24]. Although some controversy exists, the protective effect of aspirin treatment is reinforced by a recent meta-analyses study that showed a decreased overall mortality from cancer in a population using a continuous low dose of aspirin daily. This effect increased according to the treatment time [25]. This effect could be related to the potential role of the inflammation pathway in the development of PCa.

There are many studies based on the influence of statins and aspirin on PCa risk. However, even though it is common clinical practice [26], there is no report describing how a chronic treatment combining both treatments influences the risk of PCa detection. Therefore, the aim of our study was to analyze how the chronic treatment with statins, aspirin, and their combination influenced the risk of PCa detection in a cohort of patients undergoing prostate biopsy (PB) due to elevated prostate specific antigen (PSA) or abnormal digital rectal examination (DRE). We also evaluated the effect of these treatments on tumorigenic properties using different PCa cell lines, in order to corroborate clinical observations at* in vitro* level.

## 2. Materials and Methods

### 2.1. Design

We conducted a retrospective case-control study at a single academic center. All participants received detailed information about the study procedure and provided written informed consent before study entry. All procedures were in accordance with the ethical standards established in our country. Internal review board approval was not required for such a nonexperimental study.

### 2.2. Patients

Between January 2006 and December 2011, 2,408 consecutive Mediterranean men, who had been referred for prostate biopsy due to abnormal DRE and/or serum PSA levels higher than 4 ng/mL, were included in this study.

A total of 1,830 men (76%) were undergoing their first PB, while, for 578 of them, it was a repeat PB procedure. All patients were assessed by anamnesis about cardiovascular comorbidities and risk factors, life style (smoking habit and physical activity), and pharmacological treatments. A total of 1,504 men (62.5%) had not been treated with statins or aspirin, while 440 of them (18.3%) had been treated with statins, 160 (6.6%) with aspirin, and 304 (12.6%) with a combination of statins and aspirin. All of the men who received prior treatment had received it chronically for more than 1 year when this study began, and no significant differences existed among the groups, either in the initial PSA levels, in DRE results, or in the Gleason scores (see Table 1).

### 2.3. Prostatic Biopsy Technique

The PB was done as an ambulatory procedure with local anesthesia. An end-fire ultrasound transducer (Falcon 2101, B-K Medical, Inc.) and a 16-gauge automatic biopsy needle (Bard, Inc.) were used to perform the biopsy. At least 10 cores, plus 2 to 8 additional ones, according to age and prostate volume, were obtained, according to a modified Vienna nomogram [27].

### 2.4. Cell Culture

All cell lines were obtained from the American Type Culture Collection (Rockville, MD). PCa cell lines PC3 (isolated from bone metastasis of human prostate carcinoma) and LNCaP (established from a biopsy of a lymph node metastasis of hormone refractory PCa) were maintained in RPMI 1640 medium (Life Technologies, Inc., Grand Island, NY) supplemented with 10% fetal bovine serum (FBS), 2 mM l-glutamine, 100 U of penicillin/mL, 100 *μ*g of streptomycin/mL, and 0.1 mM nonessential amino acids. Cells were grown at 37°C in an atmosphere of 5% CO_2_-95% air and 99% humidity.

### 2.5. Drug Concentrations

Doses of the drugs used in this study were chosen based on previous studies by Murtola et al. [28], where, moreover, the same prostate cancer cell lines were used. Standard doses of simvastatin (STA) have been reported to obtain a peak serum value in the range of 10–100 nM; a concentration slightly above the therapeutic range had been chosen for this study (1 *μ*M) (Sigma-Aldrich). Acetylsalicylic acid (ASA) (Sigma-Aldrich) was used at a concentration of 1 mM based on common plasma salicylate levels with therapeutic doses of aspirin [29].

### 2.6. Proliferation Analysis

A number of 5 × 10^4^ cells per well were seeded on six-well plates with the appropriate medium. The cells were allowed to attach and were subsequently treated with the indicated drugs (STA, ASA, or STA + ASA) for 7 days, renewing growth medium and treatments daily. Cells were counted by using Neubauer chamber.

### 2.7. Colony Formation Assay

For the colony formation assay, cells were seeded at low density (250 cells/well in a 12-well plate) and were allowed to grow for 10 days, renewing growth medium and treatments every 2 days. Then, the cells were fixed in paraformaldehyde 4% and stained with crystal violet. Colony formation was quantified by OD reading at 590 nm after 2-hour extraction of the crystal violet stain.

### 2.8. Migration Assays

*Wound Healing*. The PC3 cells were allowed to reach confluent monolayer in 24-well plates and were incubated overnight. A straight line (wound) was then gently performed at the bottom of the dish with a 0.5 mm plastic pipette tip. Afterwards, cells were washed, incubated in medium with 2% FBS, with or without treatments, and kept in a computer controlled mini-incubator, which provided a stabilized temperature of 37°C with 95% humidity, 5% CO_2_, and optical transparency for microscopic observations. The incubator was fastened to an inverted microscope (Live Cell Imaging CellR, Olympus, Japan) to monitor cell migration. Images were taken with the 4x objective every 30 minutes at predetermined wound sites (3 sites per condition, performed in triplicate) and were analyzed using the Image J software (Wright Cell Imaging Facility, USA). Initial wound area (*μ*m^2^) and time needed to close the wound (hr) were the variables used to calculate the migration speed of the cells.

*Transwell Migration Assay.* LNCaP cells were seeded (10^5^ cells/insert) onto transwell inserts (BD Bioscience, USA) with 8 *μ*m diameter pore membranes in medium supplemented with 2% FBS in the presence or absence of treatments. After 48 hours, cells from inserts were fixed with 4% paraformaldehyde (PFA) and stained with 0.1% crystal violet. Migration was quantified by OD reading at 590 nm after 2-hour extraction of the crystal violet stain.

### 2.9. Transwell Matrigel Invasion Assay

PCa cells were seeded (10^4^/insert) onto Matrigel Invasion Chamber inserts (BD Bioscience, USA) with 8 *μ*m diameter pore membranes in triplicate in medium supplemented with 2% FBS in the presence or absence of treatments and with the lower chamber containing medium supplemented with 10% FBS. After 48 hours, the number of invading cells was determined as described above (*transwell migration assay*).

### 2.10. Western Immunoblotting

Cultured cells, washed with cold PBS, were scratched with RIPA buffer (Tris 20 mM pH 8.8, NaCl 150 mM, EDTA 5 mM, Triton X-100 1%, and protease inhibitors) and supernatant boiled at 100°C for 5 min with Laemmli buffer. The same amount of protein was resolved by 10% SDS-PAGE and transferred onto a PVDF membrane (Immobilon P^SQ^, Millipore, Massachusetts, USA). Membranes were blocked in TBS with 5% nonfat dried milk and 0.1% Tween 20 for 1 h prior to incubation at 4°C overnight with the appropriate primary antibody (1 : 200 *α*2-integrin, Santa Cruz Biotechnology, Santa Cruz, CA, USA; 1 : 400 cyclin D1, Santa Cruz Biotechnology, Santa Cruz, CA, USA; 1 : 50 vimentin, Sigma-Aldrich, Saint Louis, Missouri, USA; 1 : 2000 E-cadherin, BD Transduction Laboratories, Palo Alto, CA, USA; 1 : 1000 *α*-tubulin, Sigma-Aldrich, Saint Louis, Missouri, USA). Detection was done using horseradish-peroxidase-conjugated secondary antibodies and enhanced chemiluminescence reagents (Amersham ECL, GE Healthcare, Buckinghamshire, UK).

### 2.11. Statistical Analysis

Quantitative variables were expressed as medians, interquartile range, and range. Qualitative variables were expressed as percentages. Mann-Whitney* U* test, Wilcoxon test, and Kruskal-Wallis test were used to evaluate the significance of differences, comparing quantitative variables in two or more groups. Chi-square and Cochran tests were used to assess the normal distribution of patients, according to categorical variables and their correlation. A multivariate analysis through a binary logistic regression was performed to detect independent PC or HGPCa predictors. Odds ratios and 95% confidence interval were estimated. All analyses were calculated using SPSS v.20 (IBM, Inc.) statistical software. Statistical analysis of the cell culture data was performed by means of one-way analysis of variance (ANOVA) (Tukey's posttest).

## 3. Results

### 3.1. Observational Clinical Study

Once the final diagnosis was obtained, PCa was detected in 848 of the 2,408 patients included in this study (35.2%), 240 of whom presented with high grade PCa (HGPCa) (28.3%).

The distribution of PCa and, specifically, the HGPCa cases in relation to aspirin and statins treatments is shown in Table 2. Among the 1,504 nontreated men, PCa was detected in 552 (36.7%) without statistical differences when compared to men who were only treated with statins or those who were only treated with aspirin. In the statin-only subset, PCa was detected in 152 of 440 patients (34.5%) (*P* value = 0.879), and in the aspirin-only subset 64 PCa cases were detected (40%) (*P* value = 0.536). Interestingly, in the group of 304 patients that received concomitant treatment with statins and aspirin, PCa was detected in significantly fewer cases (26.3%) when compared to the nontreated group (*P* value = 0.003).

Regarding the rate of PCa patients who presented with HGPCa, we observed that, in the nontreated group, 24.6% of the PCa patients presented with HGPCa (136/552), whereas the aspirin group presented a significantly higher incidence rate of HGPCa, 37.5% (24/64) (*P* value = 0.034). This increase was especially noted in men who had been administrated the concomitant treatment with statins and aspirin, achieving a rate of 50% (40/80) (*P* value < 0.001). On the other hand, no statistical difference was observed in PCa patients treated with statins with HGPCa cases having a rate of 26.3% (*P* value = 0.673).

After the analysis of the above results, a binary logistic regression was conducted to evaluate if the treatment with statins and aspirin could be an independent predictor of the PCa risk and HGPCa incidence. Age, serum PSA, DRE and body mass index (BMI), statins, aspirin, and the combination between statins and aspirin treatments as well as the duration of those were included as predictive variables in this analysis (Table 3). As expected, age and serum PSA were PCa predictors. The reduction in overall PCa incidence was statistically significant for current users of both drugs. Statins and aspirin used as chronic concomitant treatment were an independent predictor factor of reduction of PCa detection risk, OR: 0.616 (95% CI: 0.467–0.812), *P* < 0.001.

Regarding the risk of high grade tumors detection, treatment with aspirin, OR: 1.835 (95% CI: 1.068–3.155), *P* = 0.028, and combined therapy, OR: 3.059 (95% CI: 1.894–4.939), *P* < 0.001, were independent factors of HGPCa. Furthermore, the length of aspirin treatment was a significant predictor of PCa, OR: 0.998 (95% CI 0.979–0.990), *P* < 0.001, while the length of statins treatment, OR: 1.005 (1.000–1.010), *P* = 0.034, and aspirin, OR: 1.033 (1.020–1.047), *P* < 0.001, was a predictor of HGPCa.

### 3.2. *In Vitro* Effects of Simvastatin, Acetylsalicylic Acid, and Their Combination

Considering the clinical observations that have been mentioned above, STA and ASA were used to treat different PCa cell lines, in order to unveil by which mechanisms the chronic treatment with these drugs significantly decreased the PCa incidence.

#### 3.2.1. Effect on the Proliferation of Prostatic Cancer Cells

**Figure 1:**
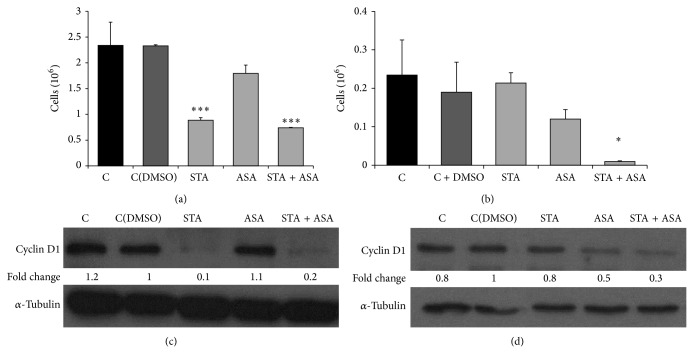
Effect of STA, ASA, and the combination of both treatments on prostate cancer cell lines proliferation. Values for proliferation are presented as number of cells (∗10^6^) after 7 days of treatment for (a) PC3 and (b) LNCaP cell lines with different conditions: nontreated cells, control (C) and control with DMSO (C(DMSO)), and cells treated with statin (STA), aspirin (ASA), and a combination of both statin and aspirin (STA + ASA) (for further details, see Section 2). The bars represent the mean ± SEM. Values that are significantly different by one-way analysis of variance (ANOVA) from the C(DMSO) group are indicated by ^*^ *P* < 0.05, ^***^ *P* < 0.001. Western blots of cyclin D1 (37 KDa) and the control α-tubulin (52 KDa) performed on total cell extracts after 7 days of treatment are presented for PC3 (c) and LNCaP (d) cell lines. Densitometry analysis using Image J software is represented below bands as fold change values (versus C(DMSO)).

Firstly, after 7 days of treatment the effects of STA, ASA, and their combination on proliferation were studied in PC3 and LNCaP PCa cell lines. The results showed a significant decrease in the proliferation rate of PC3 treated with STA (62%; *P* value = 0.001) and with the combination of both (68%; *P* value < 0.001); however, no effects were observed due to the presence of ASA in the culture media compared to control cells (cells treated only with DMSO). On the other hand, only the combination of STA and ASA was able to markedly reduce the proliferation in LNCaP cells (90%; *P* value = 0.002) (Figures 1(a)-1(b)).

To further study the effect of these treatments on the cell cycle, we analyzed the levels of cyclin D1 protein, a key protein in regulation of cell cycle. As expected, cyclin D1 showed a significant decrease in protein levels in those groups in which we also obtained a most marked decrease in cell proliferation (Figures 1(c)-1(d)).

#### 3.2.2. Effect on Colony Formation Capacity

**Figure 2:**
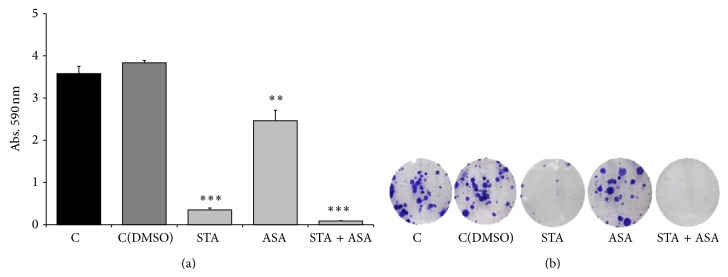
Effect of STA, ASA, and the combination of both treatments on PC3 cell line colony formation capacity. Values for colony formation capacity are presented as crystal violet absorbance/well after 10 days of treatment for PC3 cell line with different conditions: nontreated cells, control (C) and control with DMSO (C(DMSO)), and cells treated with statin (STA), aspirin (ASA), and a combination of both statin and aspirin (STA + ASA) (for further details, see Section 2). (a) The bars represent the mean ± SEM. Values that are significantly different by one-way analysis of variance (ANOVA) from the C(DMSO) group are indicated by ^**^ *P* < 0.01, ^***^ *P* < 0.001. (b) An inverted microscope with phase contrast at 10x magnification was used to take images of stained colonies.

PC3 cells were grown at low cell density (250 cells/well) in the absence or presence of the indicated concentration of STA, ASA, and the combination of both STA and ASA for 10 days. PC3 cells formed colonies, as described by others [30]; however LNCaP cells did not show the same ability to grow from so low cell density. Regarding treatment effects, long-term ASA treatment inhibited cell colony formation ability compared to control cells which had been receiving DMSO in 36% (*P* value < 0.001); furthermore, this effect was even higher in those cells treated with STA (91%; *P* value < 0.001) and the combination of both drugs (98%; *P* value < 0.001) (Figure 2).

#### 3.2.3. Effect on Migration and Invasion Capacity

In addition to cell growth and proliferation, cell mobility was studied as an important step in tumor progression; therefore, we analyzed the invasion and migration abilities of PC3 and LNCaP cells.

**Figure 3:**
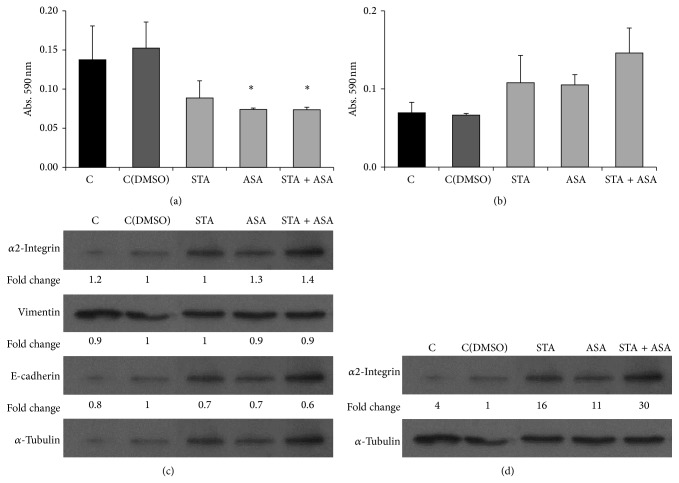
Effect of STA, ASA, and the combination of both treatments on prostate cancer cell lines invasion capacity. Values for invasion are presented as crystal violet absorbance/well for PC3 (a) and LNCaP (b) cell lines after 48 h of treatment with different conditions: nontreated cells, control (C) and control with DMSO (C(DMSO)), and cells treated with statin (STA), aspirin (ASA), and a combination of both statin and aspirin (STA + ASA) (for further details, see Section 2). The bars represent the means ± SEM. Values that are significantly different by one-way analysis of variance (ANOVA) from the control (C = nontreated) group are indicated by ^*^ *P* < 0.05 (versus C(DMSO)). Western blot of α2-integrin (150 KDa), vimentin (58 KDa), E-cadherin (120 KDa), and the control α-tubulin (52 KDa) was performed on total cell extracts after 7 days of treatment for PC3 cell line (c) and α2-integrin (150 KDa) and the control α-tubulin (52 KDa) for LNCaP (d). Densitometry analysis using Image J software is represented below bands as fold change values (versus C(DMSO)).

First, using transwell assay the invasion capacity was assessed on PC3 and LNCaP cell lines. The number of PC3 that migrated to the bottom of the transwell chambers significantly decreased compared to the nontreated group (cells with DMSO) after being treated for 48 h with ASA (48%) and STA + ASA (48%) (Figures 3(a) and 3(c)). Unexpectedly, no evident changes were found for LNCaP invasiveness (Figures 3(b) and 3(d)).

**Figure 4:**
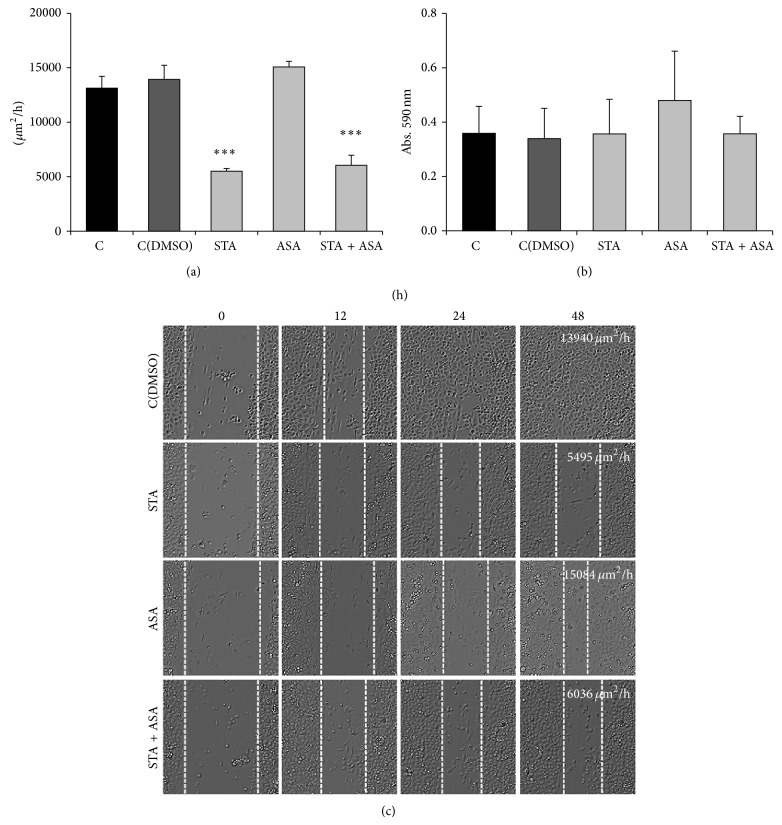
Effect of STA, ASA, and the combination of both treatments on prostate cancer cell lines invasion capacity. Values for PC3 cell line migration capacity (a) are presented as the difference between the wound-healing speed (μm^2^/h) before and after 48 h of treatment calculated using Image J software and values for LNCaP migration (b) are presented as crystal violet absorbance/well after 48 h of treatment. Both cell lines were treated with different conditions: nontreated cells, control (C) and control with DMSO (C(DMSO)), and cells treated with statin (STA), aspirin (ASA), and a combination of both statin and aspirin (STA + ASA) (for further details, see Section 2). The bars represent the mean ± SEM. Values that are significantly different by one-way analysis of variance (ANOVA) from the C(DMSO) group are indicated by ^***^ *P* < 0.001. (c) Phase contrast 10x images from migration mode during wound-healing assay at 48 h after treatment for PC3 cell line.

Second, the migration ability of both tumoral cell lines was analyzed by using wound-healing assays in PC3 line because of their ability to form a monolayer and transwell assay in LNCaP. STA and the combination of STA and ASA caused significant inhibition of PC3 migration after 48 h (61% and 57%, resp.) (Figure 4(a)) as these treatments do not allow the cells to close the wound (Figure 4(c)). On the contrary, no effect for the different treatments was observed in LNCaP cell lines (Figure 4(b)).

The lethal consequences of HGPCa are related to its metastasis to other organ sites, especially bones. Epithelial-to-mesenchymal transition (EMT) has received considerable attention as a paradigm to explain invasive and metastatic behavior during PCa progression as well as other tumors. Cells undergoing EMT lose their epithelial morphology, reorganize their cytoskeleton, and acquire a motile phenotype through the up- and downregulation of several molecules including tight and adherent junctions proteins and mesenchymal markers [31]. Bearing this in mind, the final aim of the present investigation was to see which proteins could be related to these effects on cell invasion and migration. The levels of some of those proteins, which play an important role during EMT, were analyzed (*α*2-integrin, vimentin, and E-cadherin for PC3 and *α*2-integrin for LNCaP). The results showed that, after 7 days of treatment with ASA and the combination of STA and ASA, the levels of *α*2-integrin increased slightly in PC3 cells. In the case of LNCaP, *α*2-integrin slightly increased in cells treated with ASA and increased more markedly with the combination of both treatments compared to the control cells (Figures 3(c) and 3(d)). On the other hand, no change was observed in the other proteins analyzed.

## 4. Discussion

PCa predominantly affects older men, who are likely to have comorbidities, such as cardiovascular conditions, that often indicate treatment with aspirin and statins. This find is in keeping with some authors, who have reported a significant correlation between PCa and coronary disease prevalence [5] as well as the fact that cardiovascular events are usually the first cause of death in patients with PCa [4].

In the last years some publications on this field investigated the relation between the uptake of statins and aspirin and PCa incidence; however, the impact of chronic treatment with statins and aspirin in prostate carcinogenesis is controversial. In 2008, a meta-analysis that included 6 randomized trials, 6 cohort studies, and 6 case-control studies concluded that there was neither association between statin therapy and PCa risk nor the time of treatment administration [9]. However, other studies observed a protective effect of statins on diagnosing PCa in advanced stages [9]. A similar study, where 4,202 men who had undergone PB were analyzed [32], showed that 24% of the patients that had been treated with statins at the time of PB presented a PCa detection rate of 45% compared to 42% in those patients who had not received statins. Furthermore, the Gleason score was 7 or higher in 72% of the tumors detected in chronic statins users compared to 62% in those patients not using this drug. However, the rate of patients with a Gleason score 8 or higher in radical prostatectomy specimens was 14% in both groups. This study did not take into account treatment with aspirin. With the same objective, Murtola et al. [33] analyzed the impact of statins in a series of 23,320 men (28% of them received statins) who underwent PSA screening between 1996 and 2004. The authors concluded that this treatment reduced the overall incidence of PCa, even after removing the bias produced by its effect on serum PSA. Moreover, they observed an association between time of statins therapy and the risk of PCa detection, without any relation to tumor aggressiveness. This study also monitored the use of other drugs, such as aspirin (17% of patients) and other potential confounders of PCa risk, which did not change the benefit of statins. However, the impact of combination therapy with statins and aspirin was not analyzed in this study. Aspirin has been associated with a reduced risk of PCa by some authors, while others have questioned this correlation. The chemopreventive effect of aspirin and other nonsteroidal anti-inflammatory drugs has been reported in recent data. This effect ranges from 10 to 55% [17, 18, 20, 34–37]. A recent meta-analysis that included 24 studies, 9 case controls with 5,795 men, and 15 cohorts with 31,657 patients has suggested a chemopreventive effect of aspirin on PCa [38].

With the aim of clarifying these controversial studies, we conducted a clinical observational study on 1,504 men undergoing PCa screening. Our study shows that more than a third of the population at screening of PCa was receiving some treatment to prevent cardiovascular disease. Specifically in this cohort, around 38% were chronic patients with 48% receiving statins, 18% receiving aspirin, and 34% receiving both drugs.

We observed that the detection rates of PCa in patients treated with statins or aspirin were statistically similar to the rate observed in patients who were not receiving any of these drugs. However, the detection rate of PCa observed in patients that were treated simultaneously with statins and aspirin was significantly lower (*P* value = 0.003). Furthermore, with relation to histological grade, our study suggests that chronic treatment with aspirin could be an independent predictor of HGPCa detection as well as the combination therapy with statins and aspirin. This is because, according to our analysis, there was a higher incidence of HGPCa in chronic aspirin (*P* value = 0.034) users or men receiving concomitant treatment (*P* value < 0.001).

This is not the first time that an epidemiological study has related the increase of HGPCa tumors in chronic users of aspirin [38]. However, other authors have found an inverse correlation between aspirin and HGPCa rates [39, 40]. Besides that, some studies have shown that statins are able to decrease serum PSA levels [41]. This could be considered as one possible explanation that could clarify the controversy on the effect of statins in PCa detection. The serum PSA level directly impacts the indication for PB [42, 43], and it may cause a masking bias on the possible protective effect of statins, since a population subgroup would not have biopsy criterion. In this case, the treatments may decrease the risk of PCa but also delay the diagnosis, which could explain the increased detection of HGPCa.

The present study had some limitations, which were also common in several other studies, where the effects of statins and aspirin were evaluated, for example, not being a prospective study or the effect of statins in the serum PSA hindering the evaluation of the influence of this drug in PCa. Then, there is the idea of the chronic treatment concept or not analyzing each drug dose separately, which would not allow us to draw distinct conclusions.

As a secondary objective, the tumorigenic properties of statins and aspirin on different PCa cell lines were studied, as a way to better understand our clinical observations. Thus, taking into account the results obtained* in vitro* in which the proliferation decreased dramatically as well as colony formation capacity after treating the cells with STA and ASA, we were able to hypothesize that statins and aspirin do have antitumoral effects in PCa, as other authors have observed previously [44–46]. Specifically, it could be that either aspirin treatment alone and/or simultaneous treatment may enhance the preventive effect of statins on prostate carcinogenesis at the expense of an increase in the detection of HGPCa. As we mentioned before, we have seen a direct effect of statins on proliferation, which can probably be explained by a marked decrease of cyclin D1 levels. The combination of both treatments could enhance this effect. These results, in addition to those based on the significant effect of the three treatments being higher when the cells received a concomitant treatment and on colony formation capacity, could be related to clinical observations of patients referred for PB and the incidence of PCa.

The mechanism by which statins influence prostate carcinogenesis could be mediated by the reduction of serum cholesterol and, consequently, by a decrease of intraprostatic dihydrotestosterone levels. Prostate tissues lose homeostatic control over cholesterol levels with age [47]. This effect could explain the observed differences in the results for each cell line used in our study because, as it is well known, PC3 prostate cancer cells are hormone independent and LNCaP prostate cancer cells are hormone dependent and these properties may influence the cell line response although several studies would be needed to clarify this question. The overall consequence of this cholesterol accumulation on prostate physiology is unknown, but a role for high levels of serum cholesterol in PCa incidence and progression has been suggested by a number of epidemiological and preclinical studies [10–14, 48].

In order to find an explanation for the increased incidence of HGPCa in patients treated chronically with aspirin and simultaneously with statins and aspirin, we should perform more extensive studies. However, in cancer, it is known that the expression of integrins, which are involved in cell adhesion, is frequently altered, leading to cell proliferation, migration, and metastasis. Previous studies have shown that integrin expression levels were correlated to the different stages of human cancer progression [49–51], and it has also been suggested that *α*2-integrin may be a metastasis suppressor [50]. In contrast, in PCa, *α*2-integrin was found to induce PCa cell metastasis to the bone [51, 52]. Therefore, integrin function is cell type and context dependent. Keeping in mind what we obtained, a moderate increase of these protein levels* in vitro* after ASA and concomitant treatment of STA and ASA in PC3 cell line, it could be related to those patients with a higher incidence of HGPCa. On the other hand, after performing invasion and migration assays on both PCa cell lines, the concomitant treatment seemed to present antitumorigenic effects. No changes in vimentin and E-cadherin were observed* in vitro,* which makes us think that the decrease in migration might not be related to changes in phenotype EMT. Therefore, a different approach is necessary to clarify the role of integrin and how other proteins could be implicated in the positive properties of statins and aspirin in PCa and HGPCa.

## 5. Conclusions

Although only a randomized, prospective trial that controls for patient age, comorbidities, adjuvant treatments, and dose stratification will prove the benefits of statins and aspirin treatment, the collection of all of the results presented in this study suggests that chronic concomitant treatment with statins and aspirin does have a protective effect for PCa incidence. Statins and aspirin have antitumorigenic properties, and these beneficial effects are enhanced when both treatments are administered simultaneously.

## Figures and Tables

**Table 1:** Characteristics of study groups.

	Nontreated cells	Statins	Aspirin	Statin + aspirin
Men (*n*)	1504	440	160	304
Age (years)	67	68	68	67
BMI (Kg/m^2^)	26.9	27.6	27.2	27.8
Serum PSA (ng/mL)	6.9	6.7	6.9	6.8
Positive DRE (%)	540 (36)	160 (36)	50 (31)	106 (35)
Gleason score < 7 (%)	112 (7.4)	32 (7.3)	14 (8.8)	18 (6)
Gleason score = 7 (%)	273 (18.2)	76 (17.3)	38 (23.8)	45 (14.8)
Gleason score > 7 (%)	155 (10.3)	43 (9.8)	18 (11.2)	24 (7.9)

BMI: body mass index, PSA: prostate specific antigen, and DRE: digital rectal examination.

**Table 2:** Distribution of patients according to the diagnosis of PCa and HGPCa and the treatment with statins, aspirin, and the combination of both treatments.

	PCa detection (%)	*P* value	HGPCa detection (%)	*P* value
Nontreated cells	552/1504 (37)	—	136/552 (24.6)	—
Statins	152/440 (34.5)	0.879	40/152 (26.3)	0.673
Aspirin	64/160 (40)	0.536	24/64 (37.5)	0.034
Statins + aspirin	80/304 (26.3)	0.003	40/80 (50)	<0.001

**Table 3:** Multivariate analysis of PC risk and HGPCa risk according to the treatment with statins and aspirin respect no treatment.

Treatment	PCa detection^*^	*P* value	HGPCa detection^*^	*P* value
Age (years)	1.066 (1.049–1.084)	0.001	1.100 (1.071–1.130)	0.001
BMI (Kg/m^2^)	1.018 (0.001–1.047)	0.189	0.989 (0.948–1.032)	0.616
PSA (ng/mL)	1.024 (1.008–1.041)	0.004	1.038 (1.027–1.049)	0.001
DRE (positive versus negative)	1.219 (1.028–1.428)	0.001	1.266 (0.879–1.822)	0.205
Statin (yes versus no)	0.910 (0.728–11.137)	0.430	1.092 (0.725–1.646)	0.637
Aspirin (yes versus no)	1.150 (0.824–1.604)	0.439	1.835 (1.068–3.155)	0.028
Statins + aspirin (yes versus no)	0.616 (0.467–0.812)	<0.001	3.059 (1.894–4.939)	<0.001
Time on statins (months)	0.998 (0.955–1.000)	0.063	1.005 (1.000–1.010)	0.034
Time on aspirins (months)	0.984 (0.979–0.990)	<0.001	1.033 (1.020–1.047)	<0.001

BMI: body mass index, PSA: prostate specific antigen, and DRE: digital rectal examination. ^*^Values expressed as odds ratio (95% confidence interval).
